# Clinical Report of a 17q12 Microdeletion with Additionally Unreported Clinical Features

**DOI:** 10.1155/2014/264947

**Published:** 2014-06-02

**Authors:** Jennifer L. Roberts, Stephanie K. Gandomi, Melissa Parra, Ira Lu, Chia-Ling Gau, Majed Dasouki, Merlin G. Butler

**Affiliations:** ^1^The University of Kansas Medical Center, Kansas City, KS 66160, USA; ^2^Ambry Genetics, Aliso Viejo, CA 92656, USA; ^3^King Faisal Specialist Hospital and Research Center, Riyadh 12713, Saudi Arabia

## Abstract

Copy number variations involving the 17q12 region have been associated with developmental and speech delay, autism, aggression, self-injury, biting and hitting, oppositional defiance, inappropriate language, and auditory hallucinations. We present a tall-appearing 17-year-old boy with marfanoid habitus, hypermobile joints, mild scoliosis, pectus deformity, widely spaced nipples, pes cavus, autism spectrum disorder, intellectual disability, and psychiatric manifestations including physical and verbal aggression, obsessive-compulsive behaviors, and oppositional defiance. An echocardiogram showed borderline increased aortic root size. An abdominal ultrasound revealed a small pancreas, mild splenomegaly with a 1.3 cm accessory splenule, and normal kidneys and liver. A testing panel for Marfan, aneurysm, and related disorders was negative. Subsequently, a 400 K array-based comparative genomic hybridization (aCGH) + SNP analysis was performed which identified a *de novo* suspected pathogenic deletion on chromosome 17q12 encompassing 28 genes. Despite the limited number of cases described in the literature with 17q12 rearrangements, our proband's phenotypic features both overlap and expand on previously reported cases. Since syndrome-specific DNA sequencing studies failed to provide an explanation for this patient's unusual habitus, we postulate that this case represents an expansion of the 17q12 microdeletion phenotype. Further analysis of the deleted interval is recommended for new genotype-phenotype correlations.

## 1. Introduction

The 17q12 region contains copy number variations previously reported in association with a variety of clinical findings, most frequently renal cystic disease, maturity onset diabetes of the young type 5, pancreatic atrophy, Mullerian aplasia in females, and variable cognitive involvement [1–6]. Renal cystic disease is, perhaps, the most widely reported feature resulting from the 17q12 deletion, while cognitive impairment and autism spectrum disorder have recently been associated with this deletion [2].

Moreno-De-Luca et al. [6] propose that the 17q12 deletion is among the ten most common deletions identified by microarray analysis in children with neurodevelopmental impairments. Of 15,749 patients referred for developmental delay or autism spectrum disorder in their study, 18 patients (0.11%) were found to have a 17q12 deletion. Of these 18 patients, 9 were found to have in common a 1.4 Mb deletion [34,819,670–36,203,752 bp (hg19)]. Autism spectrum disorder was found in all six male patients studied. Other common features of these patients with 17q12 deletions included macrocephaly, mild facial dysmorphism, genitourinary tract abnormalities, renal cysts, and recurrent infections of the ear, upper respiratory system, and urinary tract.

Herein, we report a patient with the 17q12 deletion using chromosomal microarray analysis presenting with autism spectrum disorder, behavioral difficulties, cognitive impairment, and joint laxity with a remarkable marfanoid body habitus, not previously described in reported cases of pathogenic copy number variations in this chromosomal region.

## 2. Subjects and Methods

### 2.1. Proband Clinical History

The proband was a 17-year-old Caucasian male. His mother and father were 40 years old, and his mother was gravida 3, para 0 at the time of conception. The 32-week gestation was complicated by preeclampsia and maternal uterine fibroids with delivery by Cesarean section. At birth, the proband weighed 2 pounds 8 ounces (1.134 kg) (3rd centile) and measured 17.5 inches (44.45 cm) (70th centile) in length [7]. He had an 8-week NICU stay with no reported respiratory difficulties. He was released home on an apnea monitor for 8 weeks with no reported apnea spells. Our proband was nonverbal until the age of 4 years. He had significant delays in gross and fine motor and social skills. He was diagnosed with autism spectrum disorder at the age of 10 years, and neuropsychological evaluation revealed a full scale IQ of 66 using the Wechsler Intelligence Scale for Children—IV. He was found to have a limited attention span but with no history of seizures and a normal electroencephalogram at the age of 15 years. A brain CT scan at the age of 15 years was normal with focal ossification noted along the dura of the left superior sagittal sinus. Attention deficit hyperactivity disorder, obsessive compulsive disorder, and autism spectrum disorder were diagnosed by a psychiatrist. Behavioral problems included irritability, physical aggression, impulsivity, and tantrums particularly when routines were disrupted. He had decreased sensitivity to pain and normal hearing and vision. Due to marfanoid body habitus, the proband received yearly echocardiograms, and early evidence of aortic root enlargement was found. CPK and homocysteine levels were performed and were normal (49 u/L and 7.3 uM, resp.). Previous surgeries included the removal of a dermoid cyst from the bridge of his nose at the age of 2 years and a partial right medial meniscectomy following a meniscus tear. The proband had a history of mildly elevated glucose levels from the age of 12 to 17 years but insulin and hemoglobin A1C levels remained within normal limits. An abdominal ultrasound was performed, and a small but otherwise normal pancreas with mild splenomegaly and a 1.3 cm accessory splenule were found. No renal abnormalities were reported.

**Figure 1:**
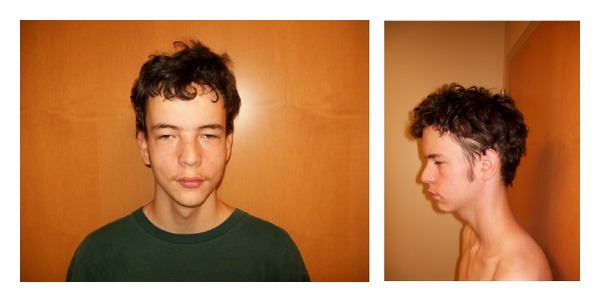
The proband presented with dysmorphic facial features, ptosis, long and narrow face, small chin, long philtrum, and high narrow palate.

**Figure 2:**
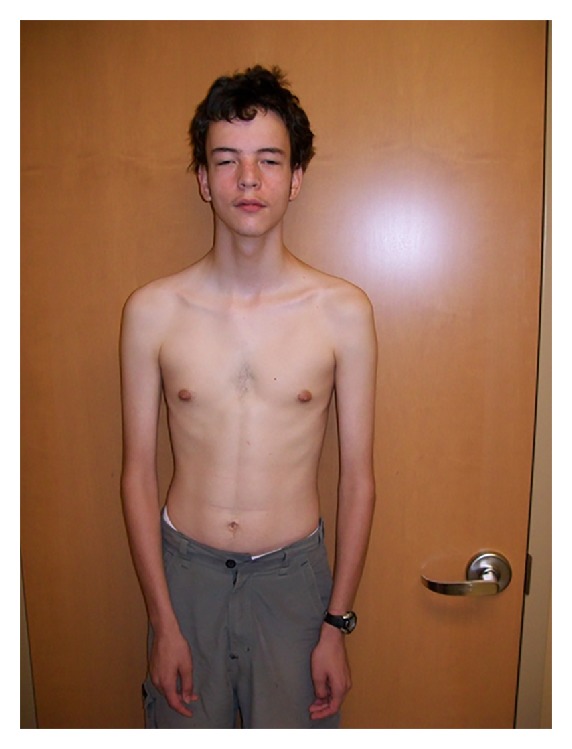
The proband presented with tall stature (90th percentile), low weight (10–25th percentile), marfanoid habitus, long fingers, hypermobile joints, mild scoliosis, and pectus deformity.

On physical exam at the age of 17 years, the proband had a height of 184 cm (89th centile), a weight of 60.4 kg (34th centile), and a head circumference of 57 cm (75th centile). He had a long- and narrow-appearing face, bilateral ptosis, relative hypertelorism, small chin, and a high, narrow palate (Figure 1). The nipples appeared widely spaced, and mild pectus deformity was observed (Figure 2). The arm span was equal to height, and no arachnodactyly was present although it had been reported in previous genetics evaluations at earlier ages. The proband had pes cavus, hypermobile small joints, and mild scoliosis.

Previous genetic testing included blood chromosome analysis which was normal (46, XY) and Marfan, aneurysm, and related disorders DNA panel, performed at Ambry Genetics (Aliso Viejo, CA), was normal. This panel included next generation sequencing of the following genes:* ACTA2*,* CBS*,* FBN1*,* FBN2*,* MYH11*,* COL3A1*,* SLC2A10*,* SMAD3*,* TGFBR1*, and* TGFBR2*.

### 2.2. Informed Consent and Sample Collection

Informed consent to perform 400 K CGH + SNP analysis was obtained from the parent as the patient was a minor. As part of the informed consent process, the diagnostic testing process was explained to the proband and his parents and questions were appropriately answered. Informed consent to perform parental FISH studies was also obtained from each parent of the proband.

### 2.3. Methodology

A blood sample was collected from the proband and sent to Ambry Genetics (Aliso Viejo, CA) for 400 K CGH + SNP analysis (Agilent Technologies, Santa Clara, CA). Genomic deoxyribonucleic acid (gDNA) was isolated from the patient's specimen using a standardized Qiagen Midi kit (Valencia, CA) and quantified by agarose gel electrophoresis. The aCGH method is based on the hybridization of fluorescently labeled patient gDNA (Cy-5) with fluorescently labeled reference DNA (Cy-3) to a 400 K oligonucleotide array (Agilent Technologies, Santa Clara, CA). Patient genomic DNA relative to the reference DNA was represented as fluorescent ratios (Cy5/Cy3), further quantified by image analysis software, and interpreted with analytical software known as BioDiscovery Nexus. Quantified results indicate each targeted-DNA sequence as loss of copy number (deletion), as gain of copy number (duplication), or as a normal copy number. Regions of homozygosity/uniparental disomy (ROH/UPD) were also analyzed.

The Ambry CMA 400 K CGH + SNP array contains 400,000 probes (~300,000 CGH probes and ~100,000 SNP probes) and covers more than 400 known genetic disorders. The array includes probes for pericentromeric and subtelomeric regions with dense probe coverage spanning 10 Mb at each subtelomere. The backbone spacing of the probes is set at an average of 13 Kb throughout the entire human genome and at 5 Kb on the X chromosome.

## 3. Results

**Figure 3:**
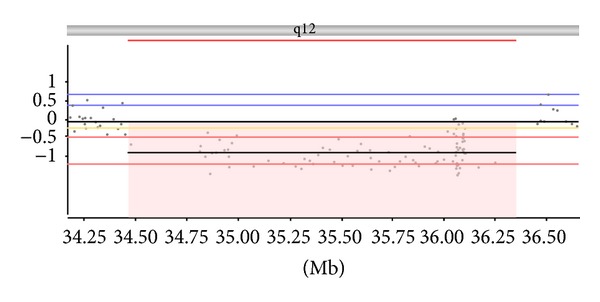
400 K CGH + SNP array result: 17q12 (34,464,879–36,352,140) × 1. 17q12 region genes:* AATF*,* ACACA*,* C17orf78*,* CCL3L1*,* CCL3L3*,* CCL4L1*,* CCL4L2*,* DDX52*,* DHRS11*,* DUSP14*,* GGNBP2*,* HNF1B*,* LHX1*,* LOC284100*,* LOC440434*,* MIR2909*,* MIRM1*,* MYO19*,* PIGW*,* SYNRG*,* TADA2A*,* TBC1D3*,* TBC1D3B*,* TBC1D3C*,* TBC1D3F*,* TBC1D3G*,* TBC1D3H*, and* ZNHIT3*.

Results of this patient's 400 K CGH + SNP analysis identified two copy number variations of interest. The first CNV identified was a loss on 1q44 (GRCh 37/hg19: 247,185,060–247,314,022 bp) encompassing 5 genes,* C1orf229*,* ZNF124*,* ZNF669*,* ZNF670*, and* ZNF670*–*ZNF695*. A review of the literature determined that deletions in this region had not been previously described and none of the genes in this interval were known to be associated with intellectual impairment or congenital abnormalities. Therefore, its clinical significance was unknown. Subsequently, parental FISH analysis revealed that this CNV was paternal in origin, and since the proband's father was clinically unaffected, it is likely benign. The second identified CNV was a loss at 17q12 (GRCh 37/hg19: 34,464,879–36,352,140 bp) which spanned a minimum size of 1.770 Mb and a maximum size of 2.005 Mb (Figure 3). Parental FISH analysis revealed that this deletion was* de novo.* It encompassed 28 genes, including* CCL3L3*,* DDX52*,* HNF1B*,* LHX1*,* TBC1D3G*, and* ZNHIT3*. Of these genes, deletion or mutation of* HNF1B* has been associated with renal cystic disease, diabetes mellitus, and liver, pancreas, and female genital tract abnormalities [2].* LHX1* is a candidate gene for the neurocognitive phenotype found in 17q12 deletion and is also expressed in the developing kidneys [1]. Other genes which may impact the phenotype of the proband include the following:* AATF* which is involved in regulation of gene transcription and cell proliferation [8],* PIGW* a complex glycolipid that anchors proteins to the cell surface [9],* TADA2A* a transcriptional adaptor [10], and* CCL3L1*,* CCL3L3*, and* CCL4L2* which are chemokines involved in inflammatory and immunoregulatory processes [11]. Chemokines are expressed in the developing brain and increased levels of chemokines in the brain, cerebral spinal fluid, and plasma have been associated with autism spectrum disorder [12]. No regions of homozygosity (loss of heterozygosity) were identified in this proband's microarray results, confirming nonconsanguineous parents.

## 4. Discussion

Several reports support the association of cognitive impairment with 17q12 deletion. In a study of 4 patients with 17q12 deletion, Nagamani et al. [5] found 3 of 4 affected individuals with developmental problems ranging from speech delay to moderate or severe intellectual disability. All patients had growth failure, and 3 of 4 patients had cystic renal disease. In addition, Dixit et al. [2] reported three patients with developmental delay of varying severity and a 17q12 deletion. These patients included a 12-year-old female with a* de novo* 1.73 Mb deletion [34,569,770–36,248,889 bp (hg 19)] detected on chromosomal microarray analysis who had speech delay and dyspraxia, mild learning difficulties, and autism spectrum disorder, a 4-year-old male with a 2.07 Mb deletion [34,611,377–36,455,391 bp (hg 19)] who had speech delay, mild hypotonia, coronal hypospadias, and dysmorphic features including telecanthus, blepharophimosis, ptosis, epicanthus inversus, anteverted nares, depressed nasal bridge, long philtrum, and small ears with overfolding of the helices, and a 7-month-old male with a 1.6 Mb maternally inherited deletion [34,611,377–36,248,889 bp (hg 19)] with a head lag and inability to sit independently. All patients had renal cysts and hypercalcemia during the neonatal period [2].

Loirat et al. [4] studied 53 patients with cystic or hyperechogenic kidneys and the 17q12 deletion was found in 3 subjects (5.6%). These three children were males with autism between the ages of 3 and 9 years with* de novo* deletions ranging from 1.49 to 1.85 Mb in size, including* HNF1B* and 19 other genes. They had early onset developmental delay, social interaction difficulties, restricted and repetitive behaviors, and communication delays. All were diagnosed with renal cysts [4].

Variable features have been reported among family members with the same 17q12 deletion. George et al. [1] reported a family in which the proband was a 7-year-old female with a 1.4 Mb deletion of the 17q12 band [34,848,922–36,249,431 bp (hg19)] including the* HNF1B* and* LHX1* genes. The proband had attention deficit hyperactivity, disruptive behavior, and learning difficulty with no renal abnormalities, while her brother was found to have the same 17q12 deletion but only mild developmental delay and bilateral renal cysts. Their mother had the same deletion with learning difficulty but no renal cysts [1].

While some reports have found an association between growth retardation and 17q12 deletion, no previous reports included tall stature or marfanoid body habitus. Hinkes et al. [3], however, reported a 38-year-old female with a 1.43 MB deletion at 17q12 [34,817,222–36,249,059 bp (hg19)] with features representing a connective tissue disorder including joint laxity, hypermobility of elbows, knees, and hips, and vascular hyperelasticity. This patient had no cognitive impairments but did have impaired renal function and uterine aplasia.

### 4.1. Implications for Clinical Practice: Expansion of the Phenotype

**Figure 4:**
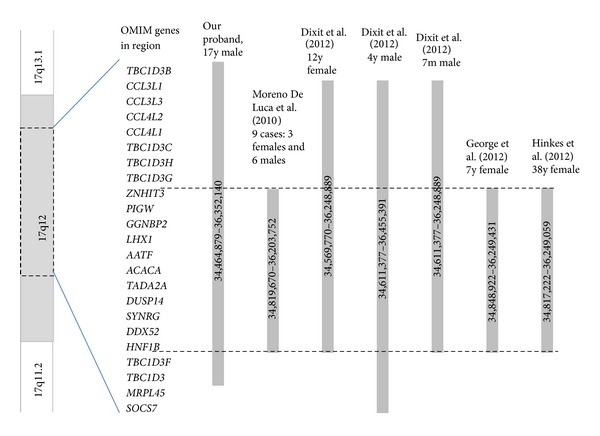
Comparison of deleted regions of 17q12. Dashed lines show the genes involved in the 17q11.2 deletion which are shared by all 7 cases. All genomic coordinates were converted to GRCh37/hg19 for comparison.

The* HNF1B* gene has been frequently reported in association with maturity onset diabetes of the young type 5 (MODY5), cystic renal disease, renal dilations, pancreatic atrophy, and liver abnormalities also seen in this deletion syndrome. Other case reports of individuals with deletions and duplications at the 17q12 critical region have included the involvement of a critical region including the* AATF*,* ACACA*,* CCL3L*,* C17orf78*,* DDX52*,* DUSP14*,* DHRS11*,* GGNBP2*,* HNF1B*,* LHX1*,* LOC284100*,* TBC1D3G*,* MRM1*,* MYO19*,* PIGW*,* SYNRG*,* TADA2A*, and* ZNHIT3* genes [1–6] (Figure 4). Associated clinical features are developmental and speech delay, significant behavioral abnormalities including aggression, self-injury, oppositional defiance, biting, hitting, and inappropriate language, and auditory hallucinations possibly indicating their role in clinical presentation [1–6] (Table 1).

Recently, Palumbo et al. [13] described a boy with a 17q12 deletion involving the* CCL4L2*,* TBC1D3H*,* TBC1D3G*,* ZNHIT3*,* MYO19*,* PIGW*,* DHRS11*,* MRM*,* LHX1*,* AATF*,* ACACA*,* TADA2A*,* DUSP14*,* SYNRG*, and* HNF1B* genes presenting with repetitive and compulsive-like behaviors, attention-deficit hyperactivity disorder, intellectual disability, language disabilities, and dysmorphic features including right posterior plagiocephaly, facial asymmetry, narrow forehead, hypotelorism, wide and fleshy auricular pavilions, protruding cheekbones, long philtrum, thin upper lip, tuft of hair on the neck, and clinodactyly of the fifth fingers [13].

The only gene contained within our proband's deletion but not within the other deletions reported in the literature prior to the recent Palumbo et al. case [13] was* TBC1D3B*. Other members of the* TBC1 domain* family including* TBC1D3C*,* TBC1D3F*,* TBC1D3G*, and* TBC1D3H* are included in our proband's deletion and in deletions described in other case reports [2, 3]. The* TBC1D3* family of oncogenes has been previously shown to enhance cellular response to epidermal growth factor [14]. Deletions of* TBC1D3B* have not been previously reported in the literature, and members of the* TBC1 domain* family are not expected to be dosage sensitive [5].

In summary, deletions of 17q12 have been reported in fewer than 100 individuals, and to our knowledge none have manifested a distinctly marfanoid habitus. We propose that our proband's unique phenotype is an expansion of the described 17q12 clinical spectrum. In support of this conclusion, it is very likely that poorly understood genes involved in our proband's deletion (such as* AATF*,* CCL3L*,* C17orf78*,* DDX52*,* DHRS11*,* GGNBP2*,* LHX1*,* LOC284100*,* TBC1D3G*,* MRM1*,* MYO19*,* SYNRG*,* TADA2A*, and* ZNHIT3*) may be responsible for these manifestations. Nevertheless, we also cannot rule out additional genetic phenomena such as variable expressivity, penetrance, and digenic inheritance due to the mutation of a second gene. Although clinical suspicion for the patient's microdeletion is responsible for his clinical presentation, further gene sequencing in this case, such as with whole clinical exome sequencing, is still warranted to rule out any additional contributing genetic factors. The authors encourage the reporting of other patients with this chromosome abnormality using microarray analysis to further delineate the genetic and clinical characteristics of pathogenic copy number alterations in this region.

## Figures and Tables

**Table 1:** Clinical phenotype in our proband and previously reported patients with 17q12 deletion.

	Our proband	Moreno-De-Luca et al. (2010) [6]	Nagamani et al. (2010) [5]	Dixit et al. (2012) [2]	Hinkes et al. (2012) [3]	George et al. (2012) [1]	Loirat et al. (2010) [4]
Clinical findings							
Mild facial dysmorphism	+	9/9	2/9	2/3	NR	NR	NR
Ptosis	+	NR	NR	1/3	NR	NR	NR
Long philtrum	+	NR	NR	2/3	NR	NR	NR
Long, narrow face	+	NR	NR	0/3	NR	NR	NR
Pectus deformity	+	NR	NR	0/3	NR	NR	NR
Joint laxity	+	NR	NR	0/3	+	NR	NR
Marfanoid body habitus	+	0/9	0/9	0/3	NR	0/2	0/3
Short stature or failure to thrive	—	1/9	3/9	1/3	NR	0/2	0/3
Kidney cysts/anomalies	—	Most*	4/9	3/3	+	1/2	3/3
Small pancreas	+	NR	NR	0/3	NR	NR	0/3
Splenomegaly	+	NR	NR	0/3	NR	NR	NR
Behavioral and cognitive features							
Autism spectrum disorder (ASD)	+	6/9	0/9	1/3	—	0/2	3/3
Developmental delay or intellectual disability	+	8/9	8/9	3/3	—	2/2	3/3
Seizures	—	0/9	2/9	0/3	—	NR	NR
Aggression	+	2/9	2/9	NR	—	NR	NR
Anxiety/disruptive behavior	+	5/9	NR	NR	—	1/2	NR
Hyperactivity	+	2/9	NR	NR	—	1/2	NR
Laboratory testing							
Neonatal hypercalcemia	NR	NR	NR	3/3	NR	NR	NR
Diabetes mellitus	—	1/9	NR	0/3	+	0/2	NR

+: feature is present, —: feature is absent, NR: not reported.

*Most of the 9 patients had kidney cystic anomalies but no specific number is given.

## References

[1] George AM, Love DR, Hayes I, Tsang B (2012). Recurrent transmission of a 17q12 microdeletion and a variable clinical spectrum. *Molecular Syndromology*.

[2] Dixit A, Patel C, Harrison R (2012). 17q12 microdeletion syndrome: three patients illustrating the phenotypic spectrum. *The American Journal of Medical Genetics A*.

[3] Hinkes B, Hilgers KF, Bolz HJ (2012). A complex microdeletion 17q12 phenotype in a patient with recurrent *de novo* membranous nephropathy. *BMC Nephrology*.

[4] Loirat C, Bellanné-Chantelot C, Husson I, Deschênes G, Guigonis V, Chabane N (2010). Autism in three patients with cystic or hyperechogenic kidneys and chromosome 17q12 deletion. *Nephrology Dialysis Transplantation*.

[5] Nagamani SCS, Erez A, Shen J (2010). Clinical spectrum associated with recurrent genomic rearrangements in chromosome 17q12. *European Journal of Human Genetics*.

[6] Moreno-De-Luca D, Mulle JG, Kaminsky EB (2010). Deletion 17q12 is a recurrent copy number variant that confers high risk of autism and schizophrenia. *The American Journal of Human Genetics*.

[7] Fenton TR (2003). A new growth chart for preterm babies: babson and Benda’s chart updated with recent data and a new format. *BMC Pediatrics*.

[8] World Wide Web (2013). *Online Mendelian Inheritance in Man*.

[9] Murakami Y, Siripanyapinyo U, Hong Y (2003). PIG-W is critical for inositol acylation but not for flipping of glycosylphosphatidylinositol-anchor. *Molecular Biology of the Cell*.

[10] Carter KC, Wang L, Shell BK, Zamir I, Berger SL, Moore PA (1997). The human transcriptional adaptor genes TADA2L and GCN5L2 colocalize to chromosome 17q12-q21 and display a similar tissue expression pattern. *Genomics*.

[11] Naruse K, Ueno M, Satoh T (1996). A YAC contig of the human CC chemokine genes clustered on chromosome 17q11.2. *Genomics*.

[12] Ashwood P, Krakowiak P, Hertz-Picciotto I, Hansen R, Pessah IN, van de Water J (2011). Associations of impaired behaviors with elevated plasma chemokines in autism spectrum disorders. *Journal of Neuroimmunology*.

[13] Palumbo P, Antona V, Palumbo O (2014). Variable phenotype in 17q12 microdeletions: clinical and molecular characterization of a new case. *Gene*.

[14] Stahl PD, Wainszelbaum MJ (2009). Human-specific genes may offer a unique window into human cell signaling. *Science Signaling*.

